# Supplementary material for: Hepatitis C prevalence in Denmark in 2016—An updated estimate using multiple national registers
Source: PLoS One. 2020 Sep 3;15(9):e0238203. doi: 10.1371/journal.pone.0238203 (PMC7470322; doi:10.1371/journal.pone.0238203)
Supplement: Attachment — Submitted filename: Response to reviewers.docx [file pone.0238203.s001.docx]

PONE-D-20-12246
Hepatitis C prevalence in Denmark in 2016

- an updated estimate using multiple national registers
PLOS ONE

***Author response to queries in bold italic***

2. In the ethics statement in the manuscript and in the online submission form, please provide additional information about the participant information used in your retrospective study.

Specifically, please ensure that you have discussed whether all data were fully anonymized before you accessed them and/or whether the IRB or ethics committee waived the requirement for informed consent.

***ANSWER: Data were fully anonymized prior to analysis. As a register-based study without contact to patients, informed consent from participants was not required, according to Danish law. This has been added to the methods section***

If patients provided informed written consent to have data from these registries/databases used in research, please include this information.

***ANSWER: Patients registered in the clinical database (DANHEP) provided written consent to have data used in research. This has been specified in the methods section.***

3. We note that you have stated that you will provide repository information for your data at acceptance. Should your manuscript be accepted for publication, we will hold it until you provide the relevant accession numbers or DOIs necessary to access your data. If you wish to make changes to your Data Availability statement, please describe these changes in your cover letter and we will update your Data Availability statement to reflect the information you provide.

***ANSWER: We have obtained accept from our data safety authority to publish the anonymized dataset. This is available as an Excel-file. (DOI: 10.5281/zenodo.3959476)***

4.Thank you for stating the following in the Financial Disclosure section:

'The study was supported by an unrestricted grant from MSD Denmark
URL: <https://www.msd.dk/home>
The funders had no role in study design, data collection and analysis, decision to publish, or preparation of the manuscript.'

We note that you received funding from a commercial source: MSD Denmark

a. Please provide an amended Competing Interests Statement that explicitly states this commercial funder, along with any other [relevant declarations](about:blank) relating to employment, consultancy, patents, products in development, marketed products, etc.

***ANSWER: we have provided a specific statement of the funding role.***

Within this Competing Interests Statement, please confirm that this does not alter your adherence to all PLOS ONE policies on sharing data and materials by including the following statement: "This does not alter our adherence to PLOS ONE policies on sharing data and materials.” (as detailed online in our guide for authors [http://journals.plos.org/plosone/s/competing-interests](about:blank)).  If there are restrictions on sharing of data and/or materials, please state these.

Please note that we cannot proceed with consideration of your article until this information has been declared.

b. Please include your amended Competing Interests Statement within your cover letter. We will change the online submission form on your behalf.

***ANSWER: we have included the statement in the cover letter.***

Please know it is PLOS ONE policy for corresponding authors to declare, on behalf of all authors, all potential competing interests for the purposes of transparency. PLOS defines a competing interest as anything that interferes with, or could reasonably be perceived as interfering with, the full and objective presentation, peer review, editorial decision-making, or publication of research or non-research articles submitted to one of the journals. Competing interests can be financial or non-financial, professional, or personal. Competing interests can arise in relationship to an organization or another person. Please follow this link to our website for more details on competing interests: [http://journals.plos.org/plosone/s/competing-interests](about:blank)


[Note: HTML markup is below. Please do not edit.]

Reviewers' comments:

Reviewer's Responses to Questions

**Comments to the Author**

1. Is the manuscript technically sound, and do the data support the conclusions?

The manuscript must describe a technically sound piece of scientific research with data that supports the conclusions. Experiments must have been conducted rigorously, with appropriate controls, replication, and sample sizes. The conclusions must be drawn appropriately based on the data presented.

Reviewer #1: Partly

Reviewer #2: No

2. Has the statistical analysis been performed appropriately and rigorously?

Reviewer #1: Yes

Reviewer #2: Yes

3. Have the authors made all data underlying the findings in their manuscript fully available?

The [PLOS Data policy](http://www.plosone.org/static/policies.action#sharing) requires authors to make all data underlying the findings described in their manuscript fully available without restriction, with rare exception (please refer to the Data Availability Statement in the manuscript PDF file). The data should be provided as part of the manuscript or its supporting information, or deposited to a public repository. For example, in addition to summary statistics, the data points behind means, medians and variance measures should be available. If there are restrictions on publicly sharing data—e.g. participant privacy or use of data from a third party—those must be specified.

Reviewer #1: Yes

Reviewer #2: Yes

4. Is the manuscript presented in an intelligible fashion and written in standard English?

PLOS ONE does not copyedit accepted manuscripts, so the language in submitted articles must be clear, correct, and unambiguous. Any typographical or grammatical errors should be corrected at revision, so please note any specific errors here.

Reviewer #1: Yes

Reviewer #2: Yes

5. Review Comments to the Author

Please use the space provided to explain your answers to the questions above. You may also include additional comments for the author, including concerns about dual publication, research ethics, or publication ethics. (Please upload your review as an attachment if it exceeds 20,000 characters)

Reviewer #1: The paper is of very poor value from the scientific point of view. The estimate of the proportion of subjects with undiagnosed CHC is affected by important biases: prisoners aren't representative of the general population,
***ANSWER As for the external validity, we agree that our samples are small and biased, but they are the only ones available in the country at the moment as no systematic HCV screening at population level is available. In addition, general population surveys also have drawbacks: most patients with chronic HCV are people with current or previous drug use and this population is usually not well captured in general population surveys (See e.g. the ECDC technical protocol for hepatitis C prevalence surveys in the general population published in March 2020. https://www.ecdc.europa.eu/en/publications-data/toolkit-support-generation-robust-estimates-hepatitis-c-prevalence). We agree that prison populations are not representative of the general population, but drug users may be tested here and we did not use the study to estimate the HCV prevalence but the undiagnosed fraction of chronic hepatitis C.***

indeed findings lack of external validity;
***ANSWER We agree that it would have been of high scientific value to perform a systematic population based survey of HCV prevalence in Denmark. However, we have not had the resources to do this. As an example testing a randomly selected population of 5000 individuals would cost 80.000€ in test kits –not including the costs of contacting and collecting the samples- and with an estimated population prevalence of 0.2% such a study would likely only identify about 10 patients with chronic HCV. In contrast the total cost of our study was less than 25.000 €. With the reservations mentioned in the discussion we find it is a cheap and feasible method to produce a national HCV prevalence estimate for Denmark.***

data from sera collected more than 20 years ago (1998-2000) are extremely out-dated:
***ANSWER: We find that the 3B samples are not outdated: There is no ongoing infection in this cohort and the few participants with HCV were infected in their youth in 60ies-80ies through drug use or nosocomial transmission prior to HCV screening introduced in the 90ies. They had not been identified in the registers since 2000 and therefor still represent the undiagnosed hepatitis C in the “pre-baby-boomer generation” that is still alive.***

Furthermore, the epidemiological data presented are obsolete because, starting from 2014, the epidemiological context has completely changed following the new direct-acting oral antiviral therapies (DAA) which, with a high sustained viral response (> 95%) and the consequent increase in treatments, have led to a faster decline of chronic hepatitis C prevalence globally.
***ANSWER: Unfortunately our data is not obsolete; In Denmark restrictions for DAA use were not removed until Nov. 2018 and few patients were treated between 2016 and 2018. Our study shows that in Denmark there will be a greater impact on HCV prevalence by contacting and treating diagnosed patients than by screening for undiagnosed HCV patients. Also importantly, our data will provide the denominator to measure the current Danish efforts to reach the WHO targets of 90% diagnosed and 80% treated.***

HCV infection is mostly asymptomatic end random population-based surveys may provide accurate figures. Several national surveys have been performed in France, Spain, Italy, and U.S.A. over the last few years. None of them has been commented in the Discussion and reported in the references.
***ANSWER: We are aware of the national population-based HCV prevalence surveys conducted in many countries -reviewed by e.g. Hofstraat et. al. 2017- and have added a reference to this in the discussion section. We have also added a short paragraph and reference to a recent HCV modelling study which found a similar proportion of diagnosed among people living with chronic HCV in Denmark to further support the data from the two external sources used in our study.***

Reviewer #2: PONE-D-20-12246

The authors used a capture recapture method to estimate the population living with HCV in Denmark in the year 2016. The concept of this study is of value and the results will contribute to the literature. However, I have some comments regarding the analysis, and the content of the study.
I have below a few comments and suggestions that may improve the readability and understandability of the article.

Major comments:
1- In the method section, the first paragraph placement is confusing (line 82-line 86). I would suggest placing this paragraph after the data sources listing since this is highlighting the indicator used for the recapture stage.
***ANSWER: We have mowed the paragraph as suggested.***

2- In the Results section, the 2nd paragraph was confusing and hard to follow. The excluded 1,292 patients from the hospital register should be mentioned in the first paragraph where the authors are listing how they ended up with the number of patients included in the analysis (Line 190-198).

***ANSWER: We have joint the two paragraphs and clarified why we don’t think the 1292 patients had hepatitis C. The bottom line is that the hospital register is not as reliable as the other registers we use, and we have removed patients who in the other registers had clear indication not to have chronic hepatitis C.***

3- Also, I don’t understand how these patients were still counted since they didn’t meet the case definition mentioned in the methods section above: "1,292 who had either negative antiHCV or HCV-RNA results and/or had chronic hepatitis B (ICD10 code 18.1) with no signs of HCV coinfection according to the other source registers."

***ANSWER: The 1.292 had the hepatitis C code in the hospital register (ICD code 18.2). However they had a later negative test for HCV in the laboratory register, or had no HCV test results and were instead positive for HBsAg or HBVDNA indicating chronic hepatitis B and suggesting a typing error in the hospital register where hepatitis B is coded as 18.0 or 18.1 We have clarified this in the text.***

Minor comments:
1- Method section line 96, the word "included" is missing: " Since 1995, it also included all 97 hospital outpatient and emergency department visits

***ANSWER: line 96 includes corrected to included”, and “it also all” changed to “it also included all”***

6. PLOS authors have the option to publish the peer review history of their article ([what does this mean?](https://journals.plos.org/plosone/s/editorial-and-peer-review-process#loc-peer-review-history)). If published, this will include your full peer review and any attached files.


If you choose “no”, your identity will remain anonymous but your review may still be made public.


**Do you want your identity to be public for this peer review?** For information about this choice, including consent withdrawal, please see our [Privacy Policy](https://www.plos.org/privacy-policy).

Reviewer #1: **Yes:** Filomena Morisco

Reviewer #2: No

[NOTE: If reviewer comments were submitted as an attachment file, they will be attached to this email and accessible via the submission site. Please log into your account, locate the manuscript record, and check for the action link "View Attachments". If this link does not appear, there are no attachment files.]

While revising your submission, please upload your figure files to the Preflight Analysis and Conversion Engine (PACE) digital diagnostic tool, <https://pacev2.apexcovantage.com/>. PACE helps ensure that figures meet PLOS requirements. To use PACE, you must first register as a user. Registration is free. Then, login and navigate to the UPLOAD tab, where you will find detailed instructions on how to use the tool. If you encounter any issues or have any questions when using PACE, please email PLOS at [figures@plos.org](mailto:figures@plos.org). Please note that Supporting Information files do not need this step.
